# Global Trends in Physical-Activity Research of Autism: Bibliometric Analysis Based on the Web of Science Database (1980–2021)

**DOI:** 10.3390/ijerph19127278

**Published:** 2022-06-14

**Authors:** Xiao-Wei Feng, Maryam Hadizadeh, Jadeera Phaik Geok Cheong

**Affiliations:** Centre for Sport and Exercise Sciences, Universiti Malaya, Kuala Lumpur 50603, Malaysia; fengxiaowei@std11.cn (X.-W.F.); jadeera@um.edu.my (J.P.G.C.)

**Keywords:** autism spectrum disorder, publication trend, Web of Science, physical activity, network analysis, bibliometrics

## Abstract

The World Health Organization has identified nervous system diseases as one of the biggest public health problems, including autism spectrum disorder (ASD). Considering the extensive benefits of physical activity (PA), the literature on the PA research of ASD has increased each year, but there is a lack of bibliometric analyses in this field. To investigate the research achievements worldwide, this paper adopts bibliometrics to analyze the trend in the academic literature on the PA research of ASD published from 1980 to 2021. The documents were retrieved from the Web of Science database, and the search strategy was to combine the keywords related to “physical activity” and “autism spectrum disorder” by using the Boolean operator tools “OR” and “AND” in the title. A total of 359 English documents were retrieved. Microsoft Excel, Data Wrapper, VOSviewer, and Biblioshiny were used for the visual analysis. We found that the number of published documents increased the fastest from 2017 to 2021, which may be due to the promulgation of the *Global Action Plan for Physical Activity 2018–2030* and the influence of COVID-19 on the world. The United States and the University of California systems are in the leading position in this field. Cooperation among countries with different levels of development will help to jointly promote the PA research progress on ASD. The focus themes include “individual effect”, “social support” and “activity dose”. The analysis of the frontier topic points out that researchers are paying increasing attention to how to improve the health and physical fitness of this group through PA. This research clearly puts forward a comprehensive overview, theme focus, and future trends in this field, which may be helpful to guide future research.

## 1. Introduction

As a neurodevelopmental disorder, autism spectrum disorder (ASD) is characterized by obstacles to social communication and interaction, and the existence of restrictive or repetitive behaviors, interests, or activities [1]. These core symptoms will last a patient’s whole life, and they cannot be better explained by intellectual disability or global developmental delay [1].

When the first childhood autism survey was conducted in Britain in the 1960s [2], the reported prevalence rate was 0.04%, and this authority remained until the end of the 20th century. The Autism and Developmental Disorders Monitoring Network (ADDM) of The Centers for Disease Control and Prevention (CDC) in the United States began to pay close attention to children with ASD in the 21st century. Since the monitoring data were first published in 2000, the prevalence rate of children with ASD has risen. In 2021, the latest data were released. Based on the data analysis in 2018, the CDC concluded that 1 out of every 44 8-year-old children was confirmed to have ASD [3]. These data refreshed the 54/1 released in 2020 [4], showing an annual growth rate of 0.42%.

In addition to the core disorder, people with ASD usually have inadequate levels of physical activity (PA) [5,6], which often leads to diseases, uncoordinated physical development, and low levels of physical fitness [7,8,9,10]. Studies have shown that PA can improve the health of children with ASD, including some psychosocial indicators [11,12,13,14], and is beneficial to behavior, communication, and academic problems, although not all cognitive processes are enhanced [13]. A large amount of the scientific literature has confirmed the importance of PA for ASD patients; if they have experienced happiness and successful PA in their early years, they may stick to these behaviors until adulthood [15].

With the deepening of research on ASD, people are paying increasing attention to the benefits of PA for ASD patients [12,16,17,18,19,20,21], and the National Standards Program (NSP) of the United States has also listed physical exercise as one of 22 emerging treatments for ASD [22]. Therefore, the academic literature reporting PA research on ASD groups is gradually increasing. As a quantitative statistical tool, bibliometric analysis is commonly used to analyze the academic literature [23]. Bibliometric analysis can visually, qualitatively, and quantitatively describe the literature in a specific field, including analysis of the citations, keyword trends, and collaboration networks among authors, institutions, and countries. Some people think that many sources may indicate that the authors or documents have attracted the attention of researchers in specific fields [24]. In recent years, some studies have been conducted from the bibliometrics perspective: research on genetic factors in ASD [25], robot intervention research [26], education research [27,28], executive function research [29], virtual reality research [30], and a scientific research overview [31] were reviewed and analyzed. Specifically, Perez-Vazquez, E et al. found, by analyzing the literature published between 2005 and 2017, that robots may help autistic children to experience real interactions in the future, but its methodological limitations will hinder the popularization of the research results in this field [26]. Additionally, Garcia, S.A found two important research directions in the field of autism education: the research on mothers of autistic children and the research on autistic teenagers. In addition, the field of education also focuses on the diagnosis and inclusion of ASD students in education centers [27]. However, no data to date are available on the bibliometric analysis of the PA of ASD patients at present. Therefore, the purpose of this study was to quantitatively and intuitively analyze the academic literature published related to PA research on ASD patients using bibliometrics.

## 2. Materials and Methods

### 2.1. Data Search Strategy

The Web of Science (WoS), as one of the most extensive databases, provides publications from high-quality scientific journals. The primary data sources in this study were A&HCI, BKCI-SSH, BKCI-S, ESCI, CPCI-SSH, CPCI-S, SCI-Expanded, and SSCI, all of which were based on the Web of Science Core Collection (WoSCC) database. The first search date was 3 January 2022, and the last literature search was conducted on 3 May 2022, to avoid deviations caused by daily updates.

**Figure 1:**
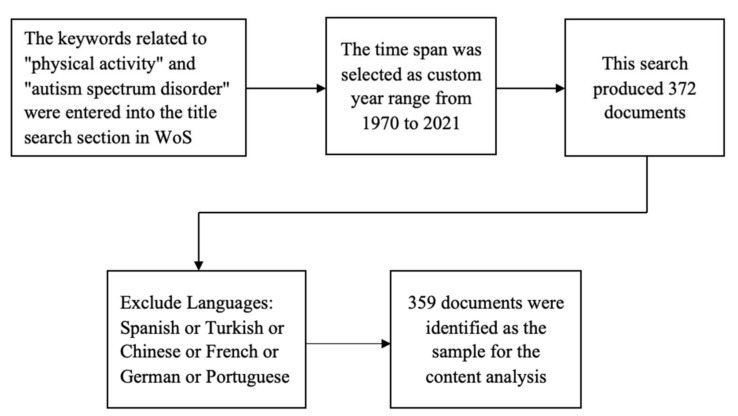
Flow chart of the document selection procedure.

Choosing appropriate keywords is very important for bibliometric analysis because it directly affects the research results [32]. After many attempts to search for different keyword combinations, and based on the published meta-analysis experience [33], our final search strategy was to combine the keywords related to “physical activity” and “autism spectrum disorder” by using the Boolean operator tools “OR” and “AND”. The specific strategies were as follows: Tl = (autis* or ASD) and Tl = (physical activity or exercise or sport or exercise gam* or active video gam*). According to the Boolean algorithm, Tl (title) refers to searching the title. Using keywords in a title search will minimize false positives and negative negatives, and keep irrelevant articles to a tolerable minimum [34]. The first Tl in the search strategy contains keywords related to ASD, while the second Tl contains keywords related to physical activity. The use of an asterisk ensures that all related words will be included in the search. The document storage time was selected as from 1 January 1970 to 31 December 2021. Initially, we obtained 372 documents. As the terms used in the search strategy were in English, the search was quasi-restricted; therefore, we excluded 13 non-English articles and finally obtained 359 papers (Figure 1).

The data used in this study came from an open database and did not involve human subjects. Therefore, the requirement for approval by the institutional review committee is exempted [35].

### 2.2. Data Analysis and Visualization

Bibliometric analysis can objectively process thousands of pieces of scientific literature, since the authors can clearly understand the publication trends through visualization [36,37]. Therefore, this article adopted the bibliometric analysis program proposed by Cobo, M.J et al. [38], which is, namely, performance analysis and scientific drawing. Performance analysis aims to measure the productivity and impact of scientific publications from the perspectives of authors, universities, and countries. We can understand scientific research structures and dynamic modes through scientific-mapping analysis.

Microsoft Excel was used to classify and execute statistical programs. Descriptive information on the authors, countries, journals, institutions, and literature was obtained through online analysis results and citation reports from the WoSCC. Journal citation reports (JCR) Science Edition 2020 was used to obtain a journal impact factor (IF), JCR, and SCImago journal rank (SJR). The raw data were exported from the WoSCC database to BibTex and CSV formats. CSV file was used to import VOSviewer [39] to create a national cooperation network and co-authorship and co-citation network of authors, and the term co-occurrence network. BibTex file was imported into Biblioshiny [40] for analysis, showing Bradford’s Law model and topic trends. Data Wrapper (https://www.datawrapper.de/tables, accessed on 3 May 2022) was used to generate a world map to show the distribution of publications and the ranking of publication categories.

Through the above tools, data analysis and visualization were realized. We mapped the annual and cumulative numbers of publications, identified the contributions and cooperation among countries, conducted a co-authorship and co-citation analysis on the authors, and analyzed terms and author keywords to discover the research focus and topic frontier.

## 3. Results

### 3.1. Analysis of Document Output

**Figure 2:**
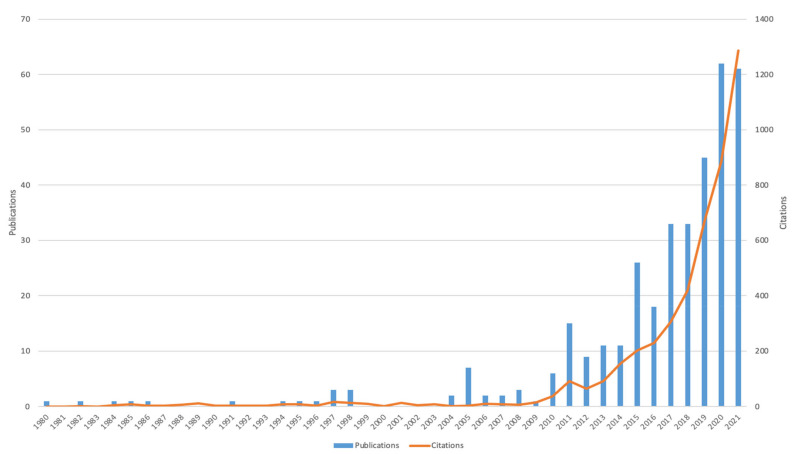
The numbers of documents and citations per year from 1980 to 2021.

Our search obtained 359 English documents, published from 1980 to 2021, regarding PA research on ASD, and they have been cited 4852 times in total, with an average of 13.52 citations per document. We found that 40 of them were highly cited; they were cited more than 40 times. In recent years, the number of studies and citations have generally shown a positive-growth trend (Figure 2). In 2020, the number of documents reached its peak (62 papers), and the number of citations reached its peak (1288 times) in 2021. The literature published in the last five years (2017–2021) accounted for more than half of the total literature in this field (234, accounting for 65.18%).

The searched literature was divided into 10 different types (Table 1). Most documents (217) were classified into the “Article” category. The highest total citations (TC) were for the type “Article” (3839), and the citations per document (CPD) of these articles was 17.69 (higher than the overall CPD of 13.52). The fewest document types belong to “Editorial Materials, Proceedings Papers, Letters, Corrections, Book Chapters, and Book reviews.” In addition, “Review” literature had the most significant number of CPDs (25.33) among all types of documents. We also found that “Meeting abstracts, Book Chapters, Letters, Corrections, Books and Book reviews” were cited the least.

**Figure 3:**
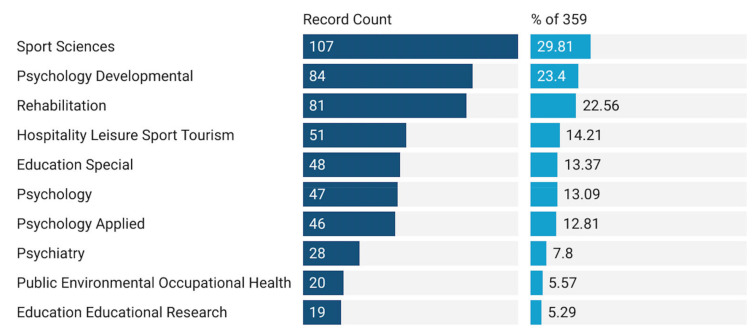
Top 10 categories of PA research of ASD in WoS.

A total of 359 pieces of literature belonged to 52 WoS subject categories. Of the 10 categories of WoS disciplines with the highest use frequency (Figure 3), the top three are Sport Sciences, Psychology Developmental, and Rehabilitation. In addition, the proportion in the Education category cannot be ignored. It is worth noting that a document may appear in more than one category.

### 3.2. Analysis of High-Yield Journals and Highly Cited Documents

**Figure 4:**
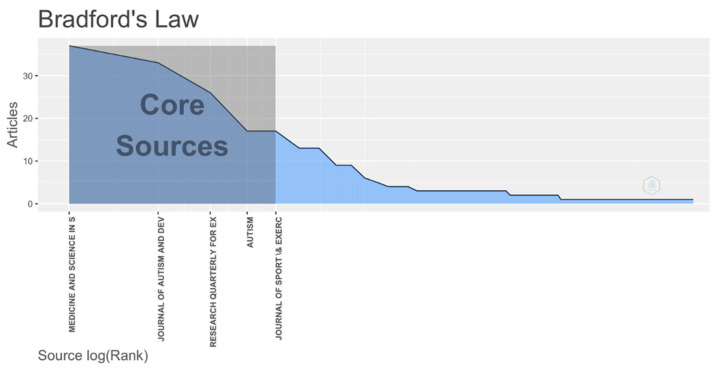
The most prominent core journals in the PA research of ASD based on Bradford’s Law.

Academic journals play a crucial role in scientific research as an essential tool for communication, as well as for the dissemination and inheritance of scientific achievements. A total of 130 scientific journals have published PA research literature on ASD, according to our analysis. Bradford’s Law states that the distribution of scientific output in relation to a particular subject is highly unequal [41,42]; we identified the top 10 most productive journals based on this law (Table 2), and found that three core journals were the most prominent: *Medicine and Science in Sports and Exercise*, *Journal of Autism and Developmental Disorders*, and *Research Quarterly for Exercise and Sport* (Figure 4). We searched the JCR database for the impact factors (2020), quartiles, and categories for these journals. Two of the top three core journals were in JCR’s Q1, indicating the high quality of scientific papers published in the JCR evaluation system. Interestingly, 6 of the 10 highly cited articles were from the United States, half of which were published in Q1 journals (Table 3), which reflects their contribution and influence in this field.

### 3.3. Analysis of Major Countries/Regions and Institutions

**Figure 5:**
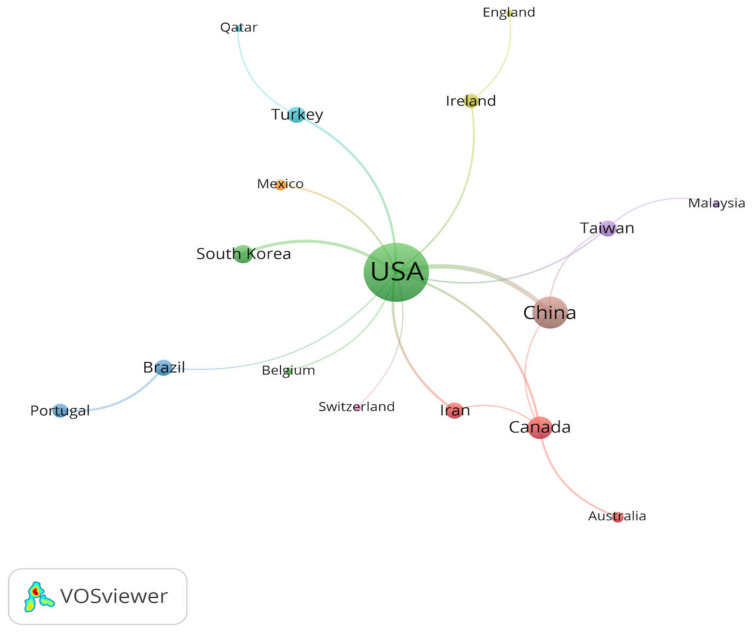
Cooperation between contributing countries/regions regarding PA research on ASD.

**Figure 6:**
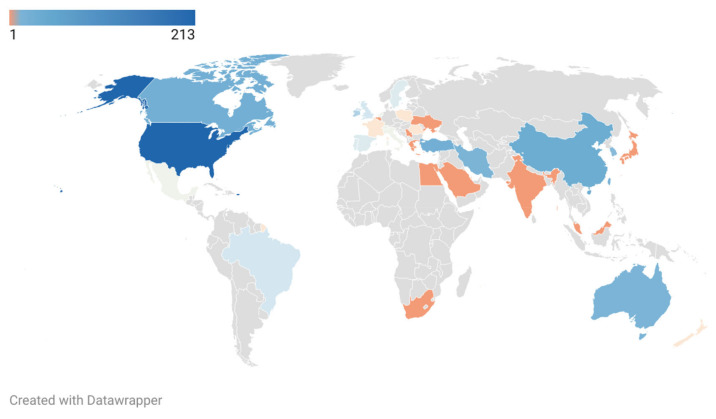
Geographical distribution of PA research in ASD publications.

Then, we evaluated the PA research status of ASD in different countries/regions. The retrieved literature was provided by 34 countries/regions, and cooperation modes were observed among major countries and regions (Figure 5). The number of publications published in a country or region is a sensitive indicator, reflecting the degree of concern and research intensity of a particular research field [25]. In this respect, the United States cooperated closely with other countries/regions and participated in most of the research, with 213 publications and an H-index of 31 (Table 4). Although the three countries/regions with the highest TC are the United States (3129), the Taiwan region (648), and Canada (363), the three countries/regions with the highest CPD are the Taiwan region (34.11), Australia (19.58), and the United States (14.69). The most active research groups mainly came from North America and Asia, while Oceania, Europe, and South America also contributed some research results (Figure 6). The main achievements of all countries and regions come from universities, and the California State University system in the United States was in the leading position in the world. The top ten most productive countries/regions included developed countries/regions and developing countries/regions, with the United States as the leader. Generally speaking, developed countries/regions have more extensive cooperation networks than developing countries/regions. Nevertheless, cooperation between countries/regions at different levels of development helps to jointly promote the PA research process of ASD from multiple sites worldwide.

### 3.4. Analysis of Main Authors

This part includes an analysis of the high-producing authors and the established co-authorship and co-citation networks of the main author.

We first analyzed the most productive authors, as shown in Table 5. The United States and the Taiwan region dominated the top 10 influential authors. Interestingly, the author who published the most articles is not from the United States, but is Chien-Yu, Pan, from the Taiwan region. He published 21 papers, which were cited 645 times, with an H-index of 11.

**Figure 7:**
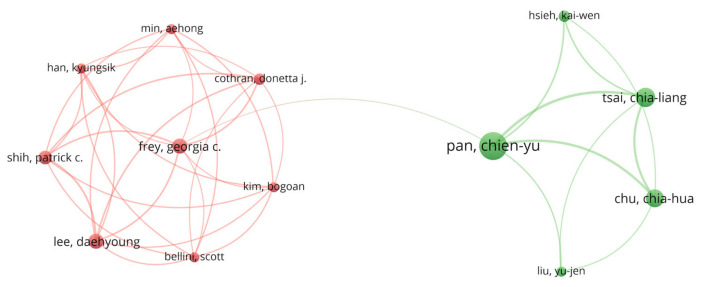
The author’s co-authorship network regarding PA research on ASD.

Then, we visually analyzed the co-authorship and co-citations of the principal authors through VOSviewer. Through a co-authorship analysis, we discovered that, out of 1013 authors, 81 authors reached the threshold of three documents, which was the minimum number of occurrences per author. The total strength of the collaboration links with other authors was derived for the 81 total authors, and a publication co-authorship network of different authors was generated (Figure 7). Considering this calculation, the complete set of associated authors consists of 13 authors from two clusters.

Each node represents the author’s name, the link represents the co-authorship between different authors, and the node size represents the number of articles that were published by each author. Chien-Yu, Pan (Taiwan), Lee, Daehyoung (USA), and Shih, Patrick C (USA) are the most influential authors in terms of co-authorship; they all have a total link strength of 25.

**Figure 8:**
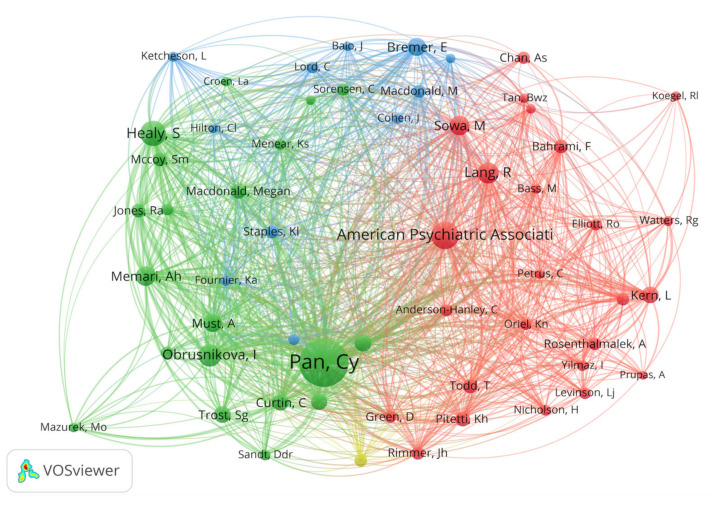
The author’s co-citation network regarding PA research on ASD.

Subsequently, we continued to conduct a co-citation analysis of the authors to explore potential partnerships. A total of 5229 co-citation authors were associated with 359 articles, and the minimum number of citations for authors was automatically set to 20, resulting in the inclusion of 57 authors. The network visualization of co-citation authors is generated by nodes and links (Figure 8). More prominent nodes and thicker links mean closer cooperation. From the co-citation network, we can see that most of the main authors are positioned at critical nodes. Chien-Yu, Pan (Taiwan) takes first place, with 342 co-citation times, followed by Healy, Sean (USA), with 112 co-citation times, and the American Psychiatric Association, with 108 co-citation times. By analyzing the most productive authors, co-authorships, and co-citations, we can identify positive and beneficial authors that are advancing PA research on ASD.

### 3.5. Focus and Frontier of PA Research of ASD

#### 3.5.1. Analysis of the Focus Themes

**Figure 9:**
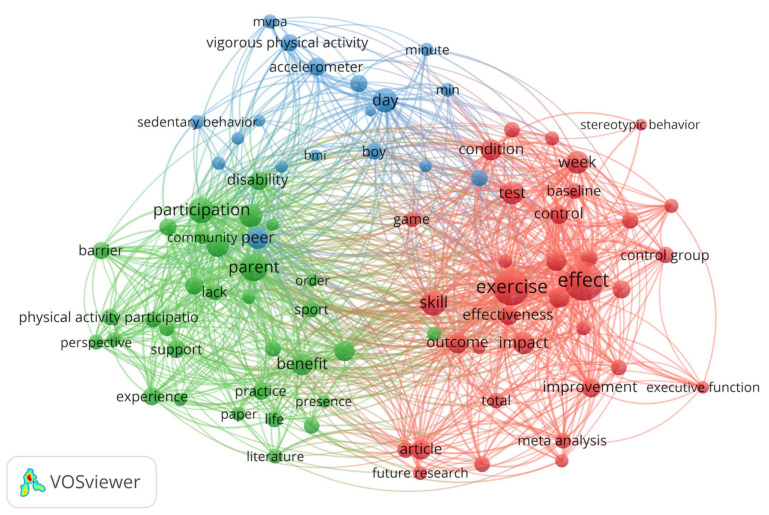
The term co-occurrence network on PA research of ASD.

To fully understand the PA research hotspots in ASD from 1980 to 2021, we continued to use VOSviewer to extract terms from the titles and abstracts of papers through natural-language-processing technology, in which terms are defined as a series of nouns or noun phrases that can be found in sentences. There were 4822 terms in 359 papers, of which 141 terms reached the automatic threshold of 10 occurrences. The relevance of each term was calculated, and 60% of the most relevant terms were selected. A total of 85 terms met these requirements. The results show that 85 entries formed three clusters and 2513 links, with a total link strength of 7287. The size of the circle or item node is proportional to the number of times a particular item appears (Figure 9). Among all the included terms, the most frequently mentioned term is “effect”, which occurs 111 times, followed by “exercise” (93 times), “parent” (56 times), “participation” (49 times), and “skill” (45 times). By classifying the terms of the three clusters, they are shown to represent: green: “individual influence”, red: “social support”, and blue: “activity dose” in the context of ASD (Table 6). These three themes reflect the research focus and achievements in the PA field of ASD over the last 41 years.

Specifically, (1) at the individual level, relevant terms such as “stereotypic behavior”, “social interaction”, and “communication” represent the core obstacles of ASD, which are the basis and primary goal of PA intervention. In this respect, since Watters, R.G., and Watters, W.E. reduced the self-stimulation behavior of a group of autistic boys through physical exercise in 1980 [52], researchers have extensively researched the effects of exercise activity on the core symptoms and related disorders of individuals with ASD (for example, see [53,54,55,56,57,58,59,60,61]). A meta-analysis in 2020 [62] examined 12 randomized controlled trials published between 2010 and 2019. The author believes that physical exercise can not only improve the motor skills of autistic children and adolescents, but also has a positive impact on social communication ability, communication ability, and related symptoms. However, no significant effect on stereotyped behavior was observed. As a feasible intervention method, these studies show that PA might provide opportunities for social development based on improving ASD individual motor skills.

(2) At the social level, relevant terms such as “family”, “parents”, “community”, “support”, and “participation” explain the primary mechanisms of PA for ASD from the perspective of social support. At present, research shows that the social support of autistic people from their typically developing peers, teachers, and parents is usually associated with increased PA [63,64,65,66]. Limitations such as the school environment (facilities and equipment), physical education content, teachers’ knowledge of teaching students with ASD, and school PA opportunities are not conducive to the PA levels of children and adolescents with ASD [44,46,65,67,68,69]. A lack of opportunities for after-school PA and negative experiences with community PA participation are also related to the lower levels of PA on weekends compared to weekdays [46,47,70,71,72].

Due to the family isolation caused by the global influence of COVID-19, researchers began to study the feasibility of providing professional support to parents through the Internet to guide children with ASD to participate in sports activities [73,74,75], thus avoiding the negative impacts of a lack of PA. Therefore, a sound social support structure, a healthy education system, and regular sports activities can help individuals to achieve healthy physical and mental development in good interpersonal relationships.

(3) At the exercise dose, relevant terms such as “day”, “accelerometer”, “mvpa”, and “vigorous physical activity” reveal the importance of exercise doses for the ASD population. Moderate-to-vigorous physical activity (MVPA) has significant benefits for health and the whole life cycle, including improved weight status, cardiovascular health, emotional health, and cognitive ability [76]. A large number of studies show that MVPA may be particularly beneficial to ASD children [13,43]. PA of a specific-intensity dose can reduce the incidence of stereotypes and repetitive behaviors [77], improve cognitive ability [78], and improve self-regulation [79], classroom performance, attention, and compliance [80], as well as social and emotional functioning [44,65]. However, further research is still needed on the best level of PA intervention for people with ASD [81]. Therefore, proper exercise intensity levels and intervention times are required to produce the best intervention effect for patients with ASD based on sound social support.

#### 3.5.2. Analysis of the Frontier Topics

Following an understanding of the focus themes, to further detect the PA research frontiers of ASD, Biblioshiny was used to visually analyze the trending topics of the authors’ keywords. Unlike the terms that were automatically extracted from the title or abstract of an article, the author keywords are taken from the list of publication keywords provided by the author. The critical role of keywords in scientific research cannot be ignored [82].

**Figure 10:**
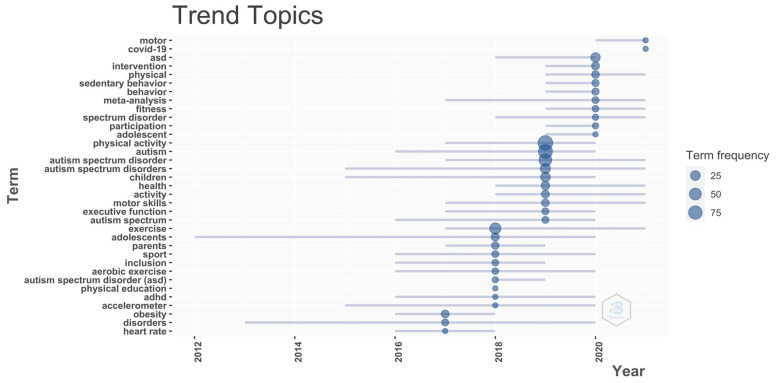
The topic trends of PA research on ASD over the last five years.

In Biblioshiny, we set the period as 2017–2021, the minimum frequency of keywords as 4, and the number of words per year as 10. This is not related to the literature but represents the subjective judgment of the authors when considering the dynamics and optimal performance of the ASD-exercise-research literature. Notably, according to the topic trend of the authors’ keywords over the last five years (Figure 10), 2020 was characterized by “ASD”, “intervention”, “physical”, “sedentary behavior”, “behavior”, “fitness”, “participation”, etc. The topic trends in 2021 were “COVID-19” and “motor”, and the prominence of these two years’ keywords may be directly related to the continuing negative impact of COVID-19 worldwide. Interestingly, “exercise”, “motor skills”, “activity”, “health”, “physical”, and “fitness” also lasted from the outbreak year to 2021, indicating that researchers noticed that developing motor skills through participating in PA and exercise may have a positive impact on the health and fitness of ASD groups.

## 4. Discussion

According to our understanding, this is the first time that a bibliometric evaluation of the physical-activity-related literature on ASD has been conducted. We aimed to obtain a global overview, as well as the focus themes and research frontiers in this field.

The results show that, since this kind of literature first appeared in 1980, the cumulative number of publications has gradually increased over the last ten years. Significantly, the literature published over the last five years (2017–2021) accounted for more than half of the total literature in this field. One possible reason for this may be that, in 2017, the World Health Organization (WHO) approved the *Global Action Plan for Physical Activity 2018–2030* (GAPPA) to prevent and control physical inactivity, promote PA, and, for the first time, include people with disabilities in the guidelines [83], which is a solid call to sport and the sports community that persons with disabilities have equal rights to PA and health. It may promote more and higher-quality research in this field [84]. On the other hand, from 2020 to 2021, the continuous influence of COVID-19 in the world led to a general lack of PA, which caused researchers to increase their attention to the health of vulnerable groups.

The survey results show that the first three categories of PA research on ASD belong to Sport Science, Psychology Developmental, and Rehabilitation. It can be seen that these three categories provide sufficient research and theoretical basis for the establishment of this topic. However, as a means of education, PA plays an important role in the education field of ASD. For example, Education Special and Education Educational Research are also among the top ten contributing categories. Students with ASD may have educational needs that other students do not have [28], and early intervention aims to reduce the symptoms of ASD patients [85] and to improve or enhance new skills [85,86]. PA has shown its related benefits to ASD patients (such as stereotyped behavior and motor skills). Therefore, it is very important for ASD people to make proper preparations for any educational program including exercise. Although the research suggests that PA research with a high-quality experimental design can help teachers, parents, and those who participate in making effective plans on a daily basis [43], interventions that are too complicated or expensive may not be easy to implement in daily life. Therefore, only by ensuring the successful transformation of the experimental research results into routine educational practice can we obtain the most effective way to encourage ASD groups to adopt an active physical lifestyle in the future, thus expanding PA’s contribution in the field of ASD education.

According to our analysis, the main journals that publish PA research on ASD are *Medicine and Science in Sports and Exercise*, *Journal of Autism and Developmental Disorders*, and *Research Quarterly for Exercise and Sport*. However, other journals are still worthy of attention. For example, although the output of *Research in Autism Spectrum Disorders* is not in the top three, the first two articles in the most cited literature are both from this journal. In addition, among the 10 most cited documents, the United States accounts for 60% of the total, and its California State University system is a leading institution in the world. Therefore, the United States is in a leading position in the PA research of ASD in the world, and its vital role in international cooperation in this field is also confirmed by the research cooperation map. This may be because of its relatively perfect ASD monitoring network and rising incidence, which has aroused the attention of researchers in this country. Interestingly, although the number of documents, total citation times, and H-index in the United States are higher than those in other countries and regions, the average citation times of each article is highest in the Taiwan region, and its H-index is second only to the United States, which indicates that the Taiwan region is also an outstanding contributor to the PA research of ASD. Due to the number of publications, there is a mismatch between the H-index and the average citation times of each article, but both of them are used to represent the publication quality and academic influence of a country or region in a certain research field.

It is also worth noting that the most active research groups mainly come from North America and Asia, while Oceania, Europe, and South America have contributed some research results, but this is not completely consistent with the epidemiological research on ASD. Generally speaking, the progress of medicine and the detection of ASD will both affect the number of related scientific publications in a country or region. With the increase in the prevalence rate, it is expected that the quality of research will be improved. However, on this issue, there is no conclusive data [29]. For example, due to the lack of universal and standardized diagnostic procedures for ASD, the total estimated prevalence of ASD in Asia is lower than that in Western countries [87]. Interestingly, five of the top 10 countries and regions are in Asia in this study. Therefore, the geographical difference in the epidemiological statistics of ASD is not the main factor hindering related research.

Although the analysis results of the main authors support the contributions of countries or regions, the United States and the Taiwan region not only have the top 10 most prolific authors in the PA research field of ASD, but they also include the most influential co-authorship and co-citation authors. However, this also further shows that the partnership between countries/regions is relatively fixed and limited. In any case, considering the impact of ASD on all races and cultures around the world, international cooperation in ASD research and publication should be encouraged and emphasized. Specifically, this should include cooperation between developed and developing countries/regions in the rehabilitation and treatment of ASD patients, which may help to promote the PA research on ASD at different levels.

Globally, with the popularization of GAPPA in various countries and the absence of PA caused by COVID-19, the PA research in the field of ASD is predicted to continue to receive attention. According to the focus themes generated by the co-occurrence of terms and the frontier topics generated based on the authors’ keywords, it is speculated that future trends in this field will continue to provide more evidence for the positive impact of PA on the core disorders and related symptoms of ASD; at the same time, this should maximize the scope of social support, especially with regard to the effectiveness of PA intervention based on the Internet, and the best dosage of exercise intervention deserves further exploration. In addition, from the perspective of public health and policy, ASD groups tend to remain sedentary [5,88], with low levels of physical activity [88]. With the increase in age, the number of individuals who participate in PA will decrease [88,89,90]. These problems have negative consequences for both short-term and long-term health. Therefore, future research will also continue to focus on the benefits of developing motor skills through participating in PA and exercises to improve the health and physical fitness of this group, which will help to reduce the huge burden borne by ASD patients and their families throughout their life.

## 5. Limitations

There are some limitations to this research. First of all, we only searched the WoS database, and not other databases, such as EMBASE, PubMed, Google Academic, and Scopus. Secondly, as English is still the most commonly used language for publishing academic literature worldwide, we only considered English publications, which led to a language deviation in this study.

## 6. Conclusions

This study conducted a bibliometric analysis of the research achievements regarding ASD in the PA field from 1980 to 2021. As a global public health issue, research in the PA field of ASD has received increasing attention over the last five years. However, only by successfully transforming the experimental research results into routine educational practice can the value of PA to ASD groups be maximized. The United States and the University of California systems play an active role in this field, but cooperation between countries/regions with different levels of development is helpful to jointly promote the progress of PA research on ASD. By introducing highly cited articles, active journals, main authors, focus themes, and research frontiers, the results may be beneficial to guide future research.

## Figures and Tables

**Table 1:** Document types of PA field in ASD.

DT	Count *	TC	CPD
Article	217	3839	17.69
Meeting Abstracts	93	9	0.1
Review Articles	33	836	25.33
Early Access	16	56	3.5
Editorial Materials	6	127	21.17
Proceedings Papers	4	34	8.5
Letters	3	7	2.33
Corrections	2	0	0
Book Chapters	1	0	0
Book Reviews	1	0	0

DT: document type, TC: total citations, CPD: citations per document. * Some documents exist in different document types simultaneously. Therefore, the total number of documents is more than 359.

**Table 2:** The top 10 productive journals on PA research of ASD.

Journal	Published Number (%)	IF 2020	SJR 2020	JCR	Categories
*Medicine and Science in Sports and Exercise*	37 (10.306%)	5.411	1.703	Q1	Physical Therapy, Sports Therapy, and Rehabilitation; Orthopedics and Sports Medicine
*Journal of Autism and Developmental Disorders*	33 (9.192%)	4.291	1.374	Q1	Developmental and Educational Psychology
*Research Quarterly for Exercise and Sport*	26 (7.242%)	2.5	0.793	Q2	Physical Therapy, Sports Therapy, and Rehabilitation; Orthopedics and Sports Medicine
*Autism*	17 (4.735%)	5.689	1.899	Q1	Developmental and Educational Psychology
*Journal of Sport and Exercise Psychology*	17 (4.735%)	3.016	0.908	Q2	Applied Psychology
*Palaestra*	13 (3.621%)	NA	NA	NA	Education and Educational Research
*Research in Autism Spectrum Disorders*	13 (3.621%)	2.881	1.040	Q2	Developmental and Educational Psychology Clinical Psychology
*Adapted Physical Activity Quarterly*	9 (2.507%)	2.929	0.618	Q2	Physical Therapy, Sports Therapy, and Rehabilitation
*International Journal of Developmental Disabilities*	9 (2.507%)	0.973	0.311	Q3	Psychiatry and Mental Health; Developmental and Educational Psychology
*Research in Developmental Disabilities*	6 (1.671%)	3.23	1.024	Q1	Developmental and Educational Psychology; Clinical Psychology

IF: impact factors, SJR: SCImago journal rank, JCR: journal citation reports, NA: not applicable.

**Table 3:** The characteristics of highly cited documents on PA research of ASD.

TC	Article Title	Journal	Published Year	Country/ Region	IF 2020	JCR
208	Physical exercise and individuals with autism spectrum disorders: A systematic review [43]	*Research in Autism Spectrum Disorders*	2010	USA	2.881	Q2
157	Effects of physical exercise on autism spectrum disorders: A meta-analysis[13]	*Research in Autism Spectrum Disorders*	2012	Netherlands	2.881	Q2
148	Physical activity patterns in youth with autism spectrum disorders [44]	*Journal of Autism and Developmental Disorders*	2006	Taiwan	4.291	Q1
126	Current perspectives on physical activity and exercise recommendations for children and adolescents with autism spectrum disorders [45]	*Physical Therapy*	2014	USA	3.021	Q1
114	Perceived barriers and facilitators of participation in after-School physical activity by children with autism spectrum disorders [46]	*Journal of Developmental and Physical Disabilities*	2011	USA	1.71	Q3
107	Comparison of physical activity between children with autism spectrum disorders and typically developing children [47]	*Autism*	2013	USA	5.689	Q1
103	Effects of water exercise swimming program on aquatic skills and social behaviors in children with autism spectrum disorders [48]	*Autism*	2010	Taiwan	5.689	Q1
102	Physical activity, dietary habits and overall health in overweight and obese children and youth with intellectual disability or autism [49]	*Research in Developmental Disabilities*	2013	New Zealand	3.23	Q1
99	The influence of vigorous versus mild exercise on autistic stereotyped behaviors [50]	*Journal of Autism and Developmental Disorders*	1984	USA	4.291	Q1
95	Barriers to physical activity in children with autism spectrum disorders: Relationship to physical activity and screen Time [51]	*Journal of Physical Activity & Health*	2015	USA	2.592	Q2

**Table 4:** Top 10 countries/regions and institutions on PA research of ASD.

Country/ Region	Articles	TC	H-Index	CPD	Top Country/Region Institutions ^1^	Top Institution Articles (%)
USA	213	3129	31	14.69	California State University System	24 (11.268%)
China	33	194	9	5.88	Chinese University of Hong Kong	8 (24.242%)
Turkey	25	103	4	4.12	Anadolu University; Erzincan Binali Yildirim University	5 (20.000%)
Canada	25	363	9	14.52	University of Toronto	7 (28.000%)
Taiwan	19	648	11	34.11	National Kaohsiung Normal University	18 (94.737%)
Australia	12	235	7	19.58	Deakin University	6 (50.000%)
Iran	11	158	6	14.36	Tehran University of Medical Sciences; University of Tehran	4 (36.364%)
South Korea	9	37	3	4.11	Ajou University; Korea University; Kyung Hee University	3 (33.333%)
Ireland	7	2	1	0.29	South East Technological University	4 (57.143%)
Brazil	5	55	3	11	Universidade Federal de Alagoas; Universidade Federal de Santa Catarina	4 (80.000%)

TC: total citations, CPD: citations per document. ^1^. Parallel institutions publish the same number.

**Table 5:** Top 10 productive authors on PA research of ASD.

Author	Articles	TC	H-Index	Country/Region
Chien-Yu, Pan	21	645	11	Taiwan
Healy, Sean	15	187	7	USA
Frey, Georgia C	11	239	3	USA
Chia-Liang, Tsai	10	216	6	Taiwan
Chia-Hua, Chu	9	169	5	Taiwan
Garcia, Jeanette M	7	90	4	USA
Haegele, Justin A	7	86	4	USA
Ketcheson, Leah	6	91	2	USA
Lee, Jihyun	6	14	2	USA
Mccoy, Stephanie M	6	101	2	USA

TC: total citations.

**Table 6:** The main terms are identified based on the clustering method.

Clusters	Cluster Terms (Occurrences ≥ 10)	Themes
Cluster 1 (Green)	35 terms: article, autistic child, baseline, change, communication, condition, control, control group, effect, effectiveness, executive function, exercise, exercise intervention, exercise program, future research, game, impact, improvement, meta-analysis, motor skill, outcome, physical exercise, pilot study, positive effect, present study, review, significant improvement, skill, social interaction, stereotypic behavior, systematic review, test, total, treatment, week.	Individual effect
Cluster 2 (Red)	32 terms: autism spectrum, barrier, benefit, challenge, community, disability, experience, factor, family, influence, interview, lack, life, literature, need, obesity, opportunity, order, paper, parent, participation, person, perspective, physical activity participation, practice, practitioner, presence, recommendation, researcher, sport, support, young adult.	Social support
Cluster 3 (Blue)	18 terms: accelerometer, adolescents, bmi, boy, brief report, comparison, day, min, minute, motivation, mvpa, pa level, peer, physical education, sedentary behavior, significant difference, vigorous physical activity, young child.	Activity dose

## Data Availability

Raw and processed data are available upon request to the corresponding author.
